# Self-Support and Loneliness Among Chinese Primary School Students: A Moderated Mediation Model

**DOI:** 10.3389/fpsyg.2021.773421

**Published:** 2022-01-18

**Authors:** Zhendong Yao, Lu Pang, Huiying Yu, Hanshi Xiao, Biao Peng

**Affiliations:** ^1^Normal College, Hunan University of Arts and Science, Changde, China; ^2^Education School of Hunan College for Preschool Education, Changde, China; ^3^School of Marxism, Guizhou Medical University, Guiyang, China; ^4^School of Public Administration, Hunan Normal University, Changsha, China

**Keywords:** self-support, loneliness, school belonging, self-esteem, primary school student

## Abstract

This study examined the effect of self-support on loneliness, the mediation effect of school belonging, and the moderation effect of self-esteem using a sample comprising 1,126 Chinese mainland primary school students, 621 are boys and 505 are girls, and their mean age was 10.51 years (*SD* = 1.63, range 8–13). Participants completed questionnaires regarding self-support, loneliness, school belonging and self-esteem. In the model hypothesis, self-support is an independent variable, loneliness is an outcome variable, school belonging is a mediating variable, and self-esteem is a regulatory variable. After controlling the demographic variables, the data were analyzed, and the results showed that: (1) self-support had a significantly negative predictive effect on loneliness; (2) the relation between self-support and loneliness was mediated by school belonging; and (3) the relation between school belonging and loneliness was moderated by self-esteem, supporting the moderated mediation model. Moderated mediation analysis further indicated that the mediated path make loneliness weaker for pupils with higher levels of self-esteem. These results revealed the formation mechanism of loneliness in primary school students and have certain enlightenment significance for the intervention of loneliness in primary school students. These results revealed the formation mechanism of loneliness among primary school students and have significant implications for interventions against loneliness in the primary school context.

## Introduction

Individuals can experience loneliness early in their lives (Vellymalay, 2010). The primary school stage is an important period for the development of childhood loneliness (Liu et al., 2013). Loneliness can be understood as the unhappiness produced by a gap between what a person has and what they lack (Perlman and Peplau, 1982). Thus, loneliness is a psychological state produced when an individual’s interpersonal relationships fail to reach the expected level, often accompanied by negative psychological experiences such as emptiness, boredom, helplessness, and depression (Kim, 2018). Essentially, loneliness is a psychological adaptation problem (Al-Yagon, 2011). In the past 30 years, the great social transformation has occurred in Chinese society, the rhythm of people’s lives has accelerated, and the adverse factors on psychology have increased. In order to cope with the rapid changes in society, the caregivers are busy with work and neglect to care for their children. The resulting problem of children’s loneliness deserves attention.

The primary school stage is a fundamental level for children’s mental development. Loneliness is an adverse psychological experience for children, and educators must pay attention to the psychological problems it causes. Several research results support this view. Studies have found that loneliness can reduce children’s mental health and threaten the normal development of their psychological function (Schinka et al., 2012). Even mild loneliness can affect physical health (Chen et al., 2019). For example, loneliness was found to partially mediate the association between the quality of social relationships and internet addiction (Guo et al., 2020), which was found to be more likely among individuals with a strong sense of loneliness. Loneliness can also lead to a decline in sleep quality (Majeno et al., 2018). Although previous studies have studied the influence of loneliness on primary school students, there are few studies on the mechanism of taking loneliness as the result variable of self-support. So, the present study aimed to examine a conceptual model in a sample of Chinese pupils in which self-support would increase school belonging. Then, the indirect association between self-support and loneliness would be moderated by self-esteem (i.e., a moderated-mediation model).

## Self-Support and Loneliness

Self-support is an indigenous Chinese personality construct (Xia et al., 2013). Independence is an important concept in western culture, which is similar to the meaning of self-support. However, self-support and independence are different personality constructs produced under different cultural, economic and political conditions. The key to the difference lies in that self-support is a dialectical personality feature, which is the result of the unity of opposites between independence and dependence (Xia and Huang, 2006b). Self-support is the process by which individuals become independent of what they used to depend on (Huang and Li, 2001).

The self-support process changes as an individual ages. The self-support behavior of primary school students can be divided into two dimensions. The domain dimension comprises self-support in the general field and self-support in a special field (academic self-support includes school life, classroom learning, homework, and research; while non-academic self-support includes action self-support, social self-support, and moral self-support). The functional dimension comprises self-determination, self-action, and self-responsibility behavior (Ling, 2006). Children’s self-support behavior is reflected in all aspects of their learning and daily lives, which in turn affects how they communicate with others and deal with problems.

Several related studies have examined the potential relationship between self-support and loneliness. Personality, as a typical individual difference variable, was found to correlated with loneliness (Jas and Ngm, 2019) and have a direct impact on loneliness (Vanhalst et al., 2013). As self-support is a kind of personality, this finding may hold for the relationship between it and loneliness as well. Moreover, the self-supporting personality was found to be closely related to interpersonal factors (Xia and Huang, 2012). A study of primary school students found that the total score for self-support was significantly positively correlated with friendship quality (Jie, 2012). According to the Social Needs Theory, everyone needs to establish social contact with others, and loneliness occurs when this need is not well met (Rotenberg and Hymel, 1999). There is a close relationship between children’s self-support behavior and self-concept: The higher the level of self-support, the more positive their self-concept (Ling, 2009). And, the cognitive style of children with high self-support levels is more inclined to field independence (Ling, 2008). Individuals with field-independent cognitive style have less interference from environmental factors, and show a more positive way in dealing with external information (Ling, 2009). Thus, self-supporting individuals are less disturbed by external factors in cognitive activities and can use their reference standards to understand and evaluate, which helps them maintain a positive state and avoid loneliness.

Hypothesis 1: There is a significantly negative correlation between primary school students’ self-support and loneliness.

## The Mediating Role of School Belonging

In the school, students are exposed to a wide variety of academic activities (Wormington et al., 2016). Students gradually form a sense of school belonging through interactions at school. A sense of school belonging is a component of student success and needs urgent attention (Arslan et al., 2020). The sense of school belonging reflects the degree of acceptance, respect, inclusion, and support felt in the school environment (Goodenow, 1993). School belonging is formed *via* not only integration but also emotional attachment to and security in the environment (Hamm and Faircloth, 2005). It reflects the student’s school performance (Singh et al., 2010).

Several studies have tested the correlation between self-support and school belonging. The self-support behavior is the embodiment of children’s problem-solving ability: The higher the level of self-support behavior, the stronger the problem-solving ability, and the better one can adapt to school, deal with interpersonal relationships and academic problems, and abide by school rules (Ling et al., 2019). Self-supporting individuals have several characteristics. Self-support is not about being dependent exclusively on oneself to solve all problems; it means being good at seeking help for survival and development needs (Xia and Huang, 2004). A study found that one of the variables most closely related to school belonging was positive personality, which is associated with good social relations (Allen et al., 2018). Students who have developed a sense of belonging to their school are more motivated to learn, more willing to participate in school activities, have better social relationships, and do better academically (Osterman, 2000).

Studies have found a significantly negative correlation between school belonging and loneliness (Yang et al., 2016). Moreover, school belonging was found to play a mediating role in the relationship between the structural characteristics of group relationships and loneliness (Chen et al., 2015). Thus, there is an association between school belonging and loneliness. This study builds a theoretical model to explain how self-support impact on loneliness through school belonging.

Hypothesis 2: School belonging plays a mediating role between self-support and loneliness.

## The Moderating Role of Self-Esteem

It is worthwhile exploring why individuals may have the same sense of school belonging but different capacities to effectively mitigate or avoid loneliness. According to Sullivan’s interpersonal relationship theory, individual social needs vary depending on the situation (Perry, 1953). Self-esteem is an psychological structure because it is a central component of an individual’s daily experience (Kernis, 2003). Self-esteem was associated with school belonging (Hernandez et al., 2017), and it can positively predict school belonging (Zhang et al., 2020). Self-esteem has been used as a social measure predicting good or bad relationships (Leary et al., 1995). Individuals whose self-esteem is threatened or who are rejected by others will often take proactive steps to try to gain a sense of self-esteem (Fang et al., 2015). So, when pupils do not adapt to school well and do not form a good sense of school belonging. Self-esteem can be adjusted as a protective factor. This will help to reduce the negative impact caused by the lack of school belonging.

Analyzing the actions taken by individuals to maintain self-esteem allows us to identify the relationship state they are in when they are attempting to get along with others. Self-esteem plays this role because restrictions on it directly affect people’s emotions and indirectly affect motivation through their effects on emotions, which then impact their whole spiritual outlook (Yang, 2003). Individuals with high self-esteem have more available resources and can fully mobilize various strategies to achieve their goals (Setliff and Marmurek, 2002). There is a significantly negative correlation between children’s self-esteem and loneliness (Lyyra et al., 2021). The satisfaction of self-esteem needs will produce self-confidence, value, strength, and ability (Maslow, 1943). In other words, feeling self-esteem is conducive to the formation of a good psychological state, which will help prevent the formation of loneliness. Therefore, this study conjectures that self-esteem plays a moderating role between school belonging and loneliness.

**FIGURE 1:**
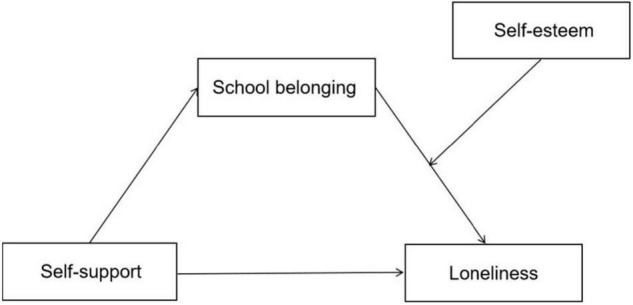
Proposed moderated-mediation model of relationship between self-support and loneliness.

Hypothesis 3: Self-esteem moderates the second stage of the indirect relationship between self-support quality and loneliness *via* school belonging. This study explores the possible mediation of school belonging between primary school students’ self-support and loneliness and the possible moderating role of self-esteem(see Figure 1).

## Materials and Methods

### Participants

Before carrying out the investigation, the research group conducted unified training to ensure that the researchers followed the principles of science and displayed efficiency and friendliness during the investigation. This study conducted the questionnaire survey in three primary schools in Mainland China. The study cohort consisted of 1,300 questionnaires, of which 1,126 valid questionnaires were received. The sample comprised 621 boys (55.20%) and 505 girls (44.80%), of whom 304 were third graders (27.00%), 290 were fourth graders (52.80%), 281 were fifth graders (25.00%), and 251 were sixth graders (22.30%). Among them, there are 506 students from only child families and 620 students from non-only child families. Their mean age was 10.51 years (*SD* = 1.63, range 8–13). The study was approved by the Ethics Committee of the Scientific Research at the first author’s institution.

## Measures

### Self-Support Behavior Questionnaire

The study’s self-support behavior questionnaire was compiled by Ling (2006). It has 62 items, measured from l (“strongly disagree”) to 5 (“strongly agree”). The total score indicates the developmental level of self-support behavior; the higher the score, the higher the level of self-support development. The questionnaire includes two dimensions: domain and function. According to the division of domain dimensions, primary school students’ self-support behavior includes the following dimensions: general domain, academic domain, daily life domain, social domain and moral domain. According to the division of functional dimensions, primary school students’ self-support behavior includes the following dimensions: self-determination, self-action, and self-responsibility. The questionnaire has a clear factor structure, good reliability, and validity. This paper mainly selects the functional dimensions as the research object. It is an effective tool for evaluating the self-support behavior of Chinese children aged 6–12 (Ling and Huang, 2006). The Cronbach’s α coefficient of sub-dimension as follow, the self-determination was 0.55, the self-action was 0.75, the self-responsibility was 0.60. The Cronbach’s α coefficient of the whole scale was 0.81.

### School Belonging Scale

The school belonging scale was compiled by Goodenow (1993) and was then revised by Pan Fada in order to create a Chinese version (Pan et al., 2011). It has 18 items, divided into three dimensions: a sense of belonging, identity, and school attachment. The scale has good reliability and validity. Seven items measure the sense of belonging (such as “the students in this school seriously adopt my opinions”; eight items measure identity) (such as “the people in this school are very friendly to me”); and three items measure school attachment (such as “in this school, I can be myself”). It is a five-point scale (1 = “never like this,” 2 = “slightly not like this,” 3 = “ordinary,” 4 = “slightly like this,” 5 = “always like this”). The Cronbach’s α coefficient of sub-dimension as follow, the sense of belonging was 0.80, the identification was 0.80, the school attachment was 0.58. The Cronbach’s α coefficient of the scale was 0.95.

### Self-Esteem Scale

The Chinese version of Rosenberg (1965) self-esteem scale was used to measure the level of self-esteem (Wang et al., 1999). It has 10 items, scored on a four-point scale (1 = very inconformity, 2 = inconformity, 3 = conformity, 4 = very conformity). The score indicates the level of self-esteem: The higher the score, the higher the self-esteem. This is a one-dimensional questionnaire. The Cronbach’s α coefficient of the scale was 0.83.

### Loneliness Scale

The children’s loneliness scale was used to assess children’s loneliness (Wang et al., 1999). The scale has 24 items, 16 of which rate loneliness and eight of which are insertions. These inserted questions help children be open and relaxed when answering the questions. It is a five-point scale (1 = “always” to 5 “never”). The higher the score, the greater the loneliness. This is a one-dimensional questionnaire. The Cronbach’s α coefficient of the scale was 0.92.

### Data Analysis

The study first examined the descriptive statistics and conducted correlation analysis of the data through SPSS 22.0. The data were then analyzed to test for the mediating role of school belonging. Finally, the data were examined to test for the moderating role of self-esteem in the mediation process. The Hayes (2013) PROCESS macro (Model 4 and 59) was evaluated during the moderated mediation model examination. Bootstrap procedures were conducted to test the moderated mediation model. Five thousand bootstrap resamples were set to calculate the 95% confidence intervals of the indirect effects in all statistical analyses. In the data analysis, the respondents’ gender, grade, and age were used as control variables.

## Results

### Preliminary Analysis

Table 1 presents the data analysis. The respondents answered the questionnaire anonymously and were informed of the confidentiality principle during the data collection in order to reduce the possible common method bias caused by the questionnaire method. Harman’s single-factor test method was used to test for the effect of program control, and the four variables were put together in an exploratory factor analysis. The results showed that 26 factors had characteristic roots greater than 1. The explanation rate of the first factor was 9.52%, which was less than 40%, indicating that the variation degree of the common method was within the acceptable range.

**TABLE 1:** Descriptive statistics and correlations for all variables.

	*M*	*SD*	1	2	3	4
1 Self-support	1.98	0.34	1			
2 School belonging	2.10	0.61	0.63**	1		
3 Self-esteem	1.91	0.46	0.56**	0.58**	1	
4 Loneliness	4.13	0.74	−0.60**	−0.66**	−0.55**	1

*N = 1,126.*

*P < 0.05 and **P < 0.01.

The mean and standard deviation of self-support, school belonging, self-esteem, loneliness, and their correlation were analyzed. As predicted, the relationships between all variables were statistically significant, consistent with the theory and previous research.

### Testing for the Mediating Effect of School Belonging

Hypothesis 2 proposed that school belonging is a mediating variable between self-support and loneliness in primary school students. To verify this hypothesis, this study used the bias-corrected bootstrap method while controlling for variables such as gender, grade, and age. The results on the mediating effect of school belonging on self-support and loneliness are presented in Table 2. The results indicate that school belonging plays a mediating role between self-support and loneliness. Self-support appears to have a negative indirect effect on loneliness through school belonging. Further, the bootstrapped 95% confidence intervals around the indirect effect do not contain zero. Therefore, the relationship between self-support and loneliness was partially mediated by school belonging.

**TABLE 2:** Mediation effect of school belonging in the relationship between self-support and loneliness.

Variable	B	*SE*	*t*	*p*
Self-support → School belonging	0.63	0.023	27.11**	0.00
School belonging → Loneliness	−0.48	0.028	−17.34**	0.00
Self-support → Loneliness	−0.30	0.028	−10.80**	0.00
Bootstrap	Effect	SE	LL95%CI	UL95%CI
Bootstrap results for indirect effect	−0.30	0.028	−0.35	−0.25

*P < 0.05 and **P < 0.01.

The above was the total score of self-support as the independent variable. In order to make the research more in-depth, next, take the three sub-dimensions of self-support as the independent variables and put them into the mediation model to test whether the model was established. The research was carried out in turn. Firstly, the independent variable self-determination was put into the mediation model. The test results showed that the mediation was established, as shown in Table 3. Then, put the self-action into the mediation model, and the test results showed that the mediation was established, as shown in Table 4. Finally, put self-responsibility into the mediation model, and the test results showed that the mediation was established, as shown in Table 5.

**TABLE 3:** Mediation effect of school belonging in the relationship between self-determination and loneliness.

Variable	B	*SE*	*t*	*P*
Self-determination → School belonging	0.44	0.27	16.65**	0.00
School belonging → Loneliness	−0.60	0.25	−24.32**	0.00
Self-determination → Loneliness	−0.40	0.027	−14.46**	0.00
Bootstrap	Effect	SE	LL95%CI	UL95%CI
Bootstrap results for indirect effect	−0.27	0.019	−0.31	−0.23

*P < 0.05 and **P < 0.01.

**TABLE 4:** Mediation effect of School belonging in the relationship between self-action and loneliness.

Variable	B	*SE*	*t*	*p*
Self-action → School belonging	0.60	0.24	25.32**	0.00
School belonging → Loneliness	−0.49	0.027	−18.34**	0.00
Self-action → Loneliness	−0.28	0.27	−10.24**	0.00
Bootstrap	Effect	SE	LL95%CI	UL95%CI
Bootstrap results for indirect effect	−0.30	0.023	−0.34	−0.25

*P < 0.05 and **P < 0.01.

**TABLE 5:** Mediation effect of School belonging in the relationship between self-responsibility and loneliness.

Variable	B	*SE*	*t*	*p*
Self-responsibility → School belonging	0.41	0.027	15.20**	0.00
School belonging → Loneliness	−0.59	0.024	−24.47**	0.00
Self-responsibility → Loneliness	−0.41	0.27	−15.10**	0.00
Bootstrap	Effect	SE	LL95%CI	UL95%CI
Bootstrap results for indirect effect	−0.24	0.021	−0.29	−0.20

*P < 0.05 and ** P < 0.01.

### Testing for Moderated Mediation Model

Because the results of self-supporting population and sub-dimensions in the mediation model are consistent, the following research uses self-supporting population as independent variable. The study found a significantly positive correlation between primary school students’ self-support and school belonging (β = 0.63, *SE* = 0.023, 95% CI [0.58, 0.67], *P* < 0.001), and the effect of school belonging on loneliness was significant negative (β = −0.039, *SE* = 0.029, 95% CI [−0.44, −0.33], *P* < 0.001). Meanwhile, self-support had a significantly negative affect on loneliness (β = −0.24, *SE* = 0.028, 95% CI [−0.29, −0.18], *P* < 0.001). In the whole model, the interaction between school belonging and self-esteem had a significantly negative affect on loneliness (β = −0.063, *SE* = 0.017, 95% CI [−0.096, −0.029], *P* < 0.001).

The bias-corrected bootstrap method indicated that the indirect effect of self-support on loneliness through school belonging was moderated by self-esteem, with the index of the moderated mediation being −0.28, 95% [−0.29,−0.20]. When the level of self-esteem was lower (i.e., 1 SD above the mean), the mediating effect of school belonging in the relationship between self-support and loneliness was significant, with the index of the mediating effect being −0.20, 95% [−0.25, −0.16]. Meanwhile, when the level of self-esteem was higher (i.e., 1 SD below the mean), a mediating effect of school belonging was found in the relationship between self-support and loneliness, with the index of the mediating effect being −0.28, 95% [−0.33, −0.23]. Therefore, when the level of self-esteem is higher, school belonging has a greater regulatory effect on loneliness.

**FIGURE 2:**
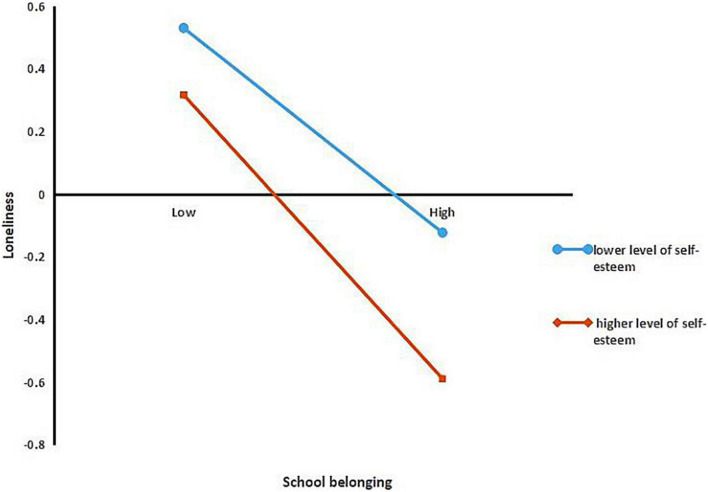
Model of test for simple slopes showing moderating influence of self-esteem in association between school belonging and loneliness.

Following the procedures suggested by Aiken and West (1991), the study examined the predictive effect of school belonging on loneliness by conducting separate examinations for the higher level (Z ≥ 1SD) and lower level (Z ≤ −1SD) of self-esteem to illustrate the nature of the moderating effect. The simple slope test indicated that, when self-esteem was higher, school belonging was associated with loneliness (β_simple_ = −0.55, *p* < 0.001); when self-esteem was lower, the effect of school belonging on loneliness was less (β_simple_ = −0.44, *p* < 0.001) (see Figure 2). These results are displayed in Table 6.

**TABLE 6:** Testing moderated mediation effect of self-esteem on self-support and loneliness.

	Model 1 (Loneliness)		Model 2 (School belonging)		Model 3 (Loneliness)	
	β	*t*	β	*t*	β	*t*
Gender	0.0090	0.20	–0.13	–2.95	–0.042	–1.00
Age	0.018	0.37	–0.13	–2.90	–0.038	–0.86
Grade	0.016	0.31	0.13	2.64	0.072	1.54
Self-support	–0.40	−14.53**	0.43	16.27**	–0.24	−8.53**
Self-esteem	–0.32	−11.63**	0.34	12.89**	–0.17	−6.43**
Self-support × Self-esteem	–0.050	−2.58*	0.027	1.45	–0.63	−3.64**
School belonging					–0.39	–13.24
School belonging × Self-esteem					–0.063	−3.66**

*P < 0.05 and **P < 0.01.

## Discussion

Loneliness is a complex psychological phenomenon that is not only related to individual psychological characteristics but is also closely linked to environmental factors (Yang et al., 2016). In other words, the occurrence of loneliness is related to the situation you are in. The social needs theory suggests the need to maintain communication and posits that a lack of interpersonal communication leads to loneliness (Terrell-Deutsch, 1999). The campus is very important for pupils. Generally, primary school students spend a great deal of time in school, except for weekends. The literature shows that personality has a direct impact on loneliness. The formation of a self-support personality is helpful for maintaining mental health, adapting to society, and achieving success (Xia and Huang, 2006a). And self-support personality helps primary school students meet their psychological needs and avoid loneliness. Self-support behavior of primary school students has three functional dimensions: self-determination, self-action and self-responsibility, which reflects a series of psychological activities of primary school students from decision to action, and then from action to responsibility. It can be seen that self-support helps primary school students to make meaningful actions and achieve their initial goals in actions. The study found a significantly negative relationship between self-support and loneliness, whereby increased self-support was associated with decreased loneliness. These results are consistent with hypothesis 1.

### Mediating Role of School Belonging

The model results supported hypothesis 2. The literature points out that personality affects the interaction between children and the environment. It will affect the psychosocial adaptation of children, reflecting the interaction between individuals and the environment (Chen et al., 2000). Self-support personality is a positive psychological quality, which helps pupils adapt to the school environment and integrate into the school family. Our study found that self-support as a distal individual factor not only had a direct predictive effect on loneliness but also indirectly influenced loneliness through school belonging.

The campus environment is important, and the experience of being discriminated against can exacerbate feelings of isolation (Majeno et al., 2018). Negative events will bring people a negative experience, and positive events will give people a positive experience. When a student receiving recognition, support, and encouragement at school and actively participating in campus activities, the student will generates a sense of belonging (Li et al., 2020). The higher sense of school belonging was found to lead to less school misbehavior (Demanet and Van Houtte, 2012). Therefore, school belonging can bring positive experiences to students. Because the self-support personality has the function of self-determination, when primary school students are in trouble, the self-supporting individual can respond in time and take action to solve the problem. So self-support serves as a resource that facilitate pupils’ school belonging, reducing the possibility of elementary school students falling into loneliness.

School belonging is a very broad concept, and it includes many aspects concerning students’ psychological attachment to their school and other people at the school. Students who think that their relationships with their teachers are negative have a lower sense of school belonging whereas those who think that their relationships with teachers are positive have a higher sense of school belonging (Slaten et al., 2016). When primary school students enter school, they need to study every class carefully, complete the homework carefully, and live in harmony with classmates and teachers. All of these are inseparable from self-support personality. Without self-support personality as basis, primary school students will find it difficult to perform their duties and become popular children. The special fields of primary school students’ self-support include two parts: academic self-support and non-academic self-support. The content of these two parts is to complete the academic tasks assigned by the teachers for primary school students and the important psychological quality favored by the teachers. Therefore, pupils who show more self-support behavior can better adapt to the school, integrate into the collective and form a sense of belonging to the school. r The generation of loneliness is directly related to the satisfaction of psychological needs. After the psychological needs are met, the generation of loneliness will be reduced. The generation of school belonging lays a psychological foundation for the satisfaction of pupils’ psychological needs. School is a social environment children enter. It is an important place for children’s socialization, and the development of self-support is conducive to the satisfaction of school belonging. Increasing students’ sense of school belonging can also improve their executive function (Dixson and Scalcucci, 2021). Pupils with strong executive function will show more outstanding achievements in dealing with various things, and the achievement of these outstanding achievements can well meet the psychological needs of pupils. Thus, there is a positive social link between pupil and school belonging, which can help primary school students avoid or reduce loneliness.

### Moderating Role of Self-Esteem

The results support hypothesis 3 by indicating that self-esteem moderated the second stage of the indirect relationship between self-support quality and loneliness *via* school belonging. School belonging negatively predicted loneliness, and self-esteem had a moderating effect between them. Compared to those pupils with lower school belonging, those with high self-esteem who form self-support personality have the relationship between school belonging and less loneliness strengthened.

Individuals with a higher sense of school belonging and higher self-esteem tend to have a lower sense of loneliness. Individuals with a high level of self-esteem who lack a strong sense of school belonging and are rejected in school can avoid negative consequences. Because self-esteem serves as a buffer that helps individuals to avoid being hurt by negative events or to recover from them (Luo and Cai, 2012). The research also shows that individuals with high self-esteem usually have better interpersonal relationships (Baumeister et al., 2003), whereas those with lower self-esteem are more dissatisfied in their interpersonal relationships (Wang et al., 2018). Self-esteem has become a very good regulator, providing a guarantee for pupils to meet setbacks and overcome difficulties. When pupils are in trouble, self-esteem will urge them to try their best to break away from the trouble and return to their former state. Because the life satisfaction of a person has a high level of self-esteem may not be negatively impacted by social exclusion (Arslan, 2019). So, self-esteem plays a positive role in life satisfaction (Moksnes and Espnes, 2013). Thus, self-esteem is a protective factor for pupils. In this sense, regulator by high-level self-esteem acts as a more adaptive form of self-management that can replace negative state, such as loneliness. Hence, our study supports hypothesis 3.

In summary, this study identified a significant moderatedmediation model that explained the effect of self-support on pupils’ loneliness, which responded to the reasons for the formation of loneliness. The model we propose provides research on the relationship between self-support and loneliness in primary school students, and provides corresponding empirical evidence for the theory of experiential avoidance of loneliness, and provides some potential methods to enhance the benefits of self-support to students. This study revealed that the main mediating mechanism of school belonging and primary school students’ self-esteem are a variable to explain the heterogeneity of the relationship between self-support and primary school students’ loneliness.

### Limitations and Practical Implications

This study has several limitations. First, it was a cross-sectional study and did not reveal changes in variables over time. Future research should conduct tracking studies to reveal the paths of the models. Second, the study used the self-report method. Although this method is common, it may collect non-true answers. Future research should consider collecting data through multiple channels and methods. Third, in the collection of demographic information, the collection of parents’ economic counterpoint, family type and other related information is ignored. Further studies should investigate these information. Finally, this study’s sample comprised Chinese pupils. Future studies should expand the sample’s scope.

Despite its limitations, this study takes an important step toward understanding how self-support is related to loneliness. The study found that the formation of loneliness among pupils is determined by multiple factors rather than one single factor. Thus, educators should analyze the causes of pupils’ loneliness in terms of multiple factors, which would help them make better judgments. Moreover, the study’s results on self-esteem can assist in planning interventions to deal with loneliness among primary school children. For example, interventions can be applied to reduce the negative effects of loneliness for individuals with low levels of self-support and school belonging by enhancing their self-esteem.

School leaders should pay attention to the formation and development of students’ school belonging (Koçak, 2021). The school should be a caring community, and the staff should feel responsible for the cultivation of every student (Demanet and Van Houtte, 2012). At the same time, education should foster the cultivation of self-esteem, especially for those pupils who lack self-support and school belonging, by helping students to improve their self-esteem and relieve their loneliness.

## Data Availability Statement

The raw data supporting the conclusions of this article will be made available by the authors, without undue reservation.

## Ethics Statement

The study was approved by the Ethics Committee of the Scientific Research at Hunan University of Arts and Science. Written informed consent to participate in this study was provided by the participants’ legal guardian/next of kin.

## Author Contributions

ZY designed the study and wrote manuscript. LP collected the data and wrote manuscript. HY designed the study and revised manuscript. HX designed the study and collected the data. BP analyzed the data. All authors contributed to the article and approved the submitted version.

## Conflict of Interest

The authors declare that the research was conducted in the absence of any commercial or financial relationships that could be construed as a potential conflict of interest.

## Publisher’s Note

All claims expressed in this article are solely those of the authors and do not necessarily represent those of their affiliated organizations, or those of the publisher, the editors and the reviewers. Any product that may be evaluated in this article, or claim that may be made by its manufacturer, is not guaranteed or endorsed by the publisher.
